# Pathogenic and Epiphenomenal Anti-DNA Antibodies in SLE

**DOI:** 10.4061/2010/462841

**Published:** 2010-07-20

**Authors:** Mirjana Pavlovic, Anna Kats, Michelle Cavallo, Ran Chen, James X. Hartmann, Yehuda Shoenfeld

**Affiliations:** ^1^Department of Computer and Electrical Engineering and Computer Science, Florida Atlantic University, Boca Raton, FL 33431, USA; ^2^Department of Biology, Florida Atlantic University, Boca Raton, FL 33431, USA; ^3^Center for Autoimmune Diseases, Sheba Medical Center, Tel Hashomer 52621, Israel

## Abstract

The discoveries of natural and the development of manufactured highly efficient catalytic antibodies (abzymes) opens the door to many practical applications. One of the most fascinating is the use of such antibodies in human therapy and prevention (vaccination), of cancer, AIDS, autoimmune diseases. A special entity of naturally occurring DNA hydrolytic anti-DNA antibodies is emerging within past decades linked to autoimmune and lymphoproliferative disorders, such as systemic lupus erythematosus (SLE), multiple sclerosis (MS), Sjogren Syndrome (SS), B - Chronic lymphocytic leucosis (B-CLL), and Multiple Myeloma (MM). The origin of the antibodies is unknown. The underlying mechanisms of these activities are suggested to be penetration into the living cells and translocation in the nucleus, with recognition of the specific binding sites at particular (ss or ds) DNA. There are controversies in the literature whether hydrolysis is a sequence-specific event. The interplay between anti-DNA antibodies and DNA is not yet elucidated. This molecular “twist” also suggests that anti-DNA antibodies with DNA hydrolytic capacity could be the organism's immune response to a microbial attack, with microbial DNA, or specific genes within microbial DNA sequence, as a target for neutralization. The catalytic antibody-based approach can become a key tool in selective chemotherapeutic strategies.

## 1. Introduction: Historical Notes

In 1957 [1], Ceppelini et al. reported that components present in the sera from SLE patients were reactive with DNA. These components were subsequently identified as antibodies, and a broad spectrum of methods have been developed in order to improve detection, characterization, and quantification of anti-DNA autoantibodies [2, 3]. These methods were initially employed during investigations into the role of these antibodies in SLE, but later research revealed their occurrence in other autoimmune diseases. Yet, anti-dsDNA autoantibodies are still considered by clinicians the hallmark of lupus disease. Recently, an interest in autoantibodies produced against ssDNA occurred in both clinical [4–8] and experimental studies [9–11]. Key questions regarding their presence and role in the development of SLE and other autoimmune diseases remain. Recent studies on the structure, function, and pathogenicity of both types of autoantibodies revealed their dual function: hydrolysis of DNA and cytotoxicity toward tumor cell lines [10, 12–14]. These functional features as both enzymes and cytotoxic antibodies have recently been of keen interest in the clinical arena [15, 16]. An understanding of the duality of these unique antibodies may shed light on the mechanisms of their pathogenicity in SLE and other autoimmune diseases. 

## 2. Anti-dsDNA Antibodies versus Anti-ssDNA Antibodies

### 2.1. Anti-DNA Antibodies and Their Correlation with SLE Pathogenesis

Systemic lupus erythematosus (SLE) is a chronic, potentially fatal autoimmune disease characterized by exacerbations and remissions with various clinical manifestations affecting multiple organ systems, including the skin, kidney, joints, cardiovascular, and nervous system. The hallmark of systemic lupus erythematosus is the production of an array of IgG and IgM autoantibodies directed against one or more nuclear components, the most frequent of which are double stranded (ds) DNA and/or single stranded (ss) DNA. Both anti-ssDNA and anti-dsDNA are involved in disease development and have been eluted from the kidneys of both experimental murine models and SLE patients [17]. Clinicians consider anti-dsDNA autoantibodies to be fairly disease specific, while anti-ssDNA to be nonspecific. The reasons for the difference in disease specificity are that there is a test sensitivity of 60% for the anti-dsDNA antibodies while the variability is 30%–70% for the latter [18]. The level of anti-DNA antibodies varies in different SLE patients' plasma, with high levels of anti-ssDNA and/or anti-dsDNA antibodies being associated with the flare of symptoms [4, 5]. Consequently, the level of anti-DNA antibodies in patients' sera is used to monitor disease activity and progression [4–6].

The precise mechanisms leading to anti-DNA antibody production remain unknown. The subsets of B-cell producers vary according to different authors [19–21]. Furthermore, the mechanisms of the pathogenicity of anti-DNA antibodies and the immune complexes in SLE are disputed. Part of the pathogenicity may be due to direct hydrolytic and cytotoxic activity of the anti-DNA antibodies upon the cells of different tissues and organs that are affected in the disease [18]. Pathogenic anti-DNA antibodies are able to interact at the tissue level with alpha actinin in the glomeruli of kidney. 

**Figure 1:**
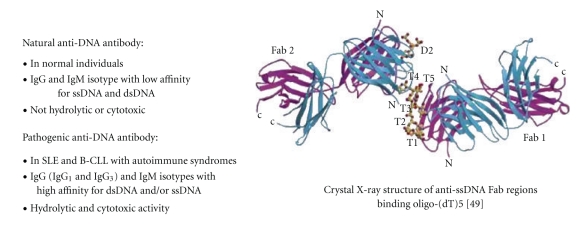
Differences between natural and pathogenic anti-DNA autoantibodies.

At the cellular level, the antibodies have been shown to react with various cell surface proteins (e.g., myosin 1), presumably allowing their penetration into the cell [22–25]. Upon entry into the cell, anti-DNA antibody is translocated into the nucleus where it binds to DNA and consequently hydrolyzes it. An alternative mechanism by which anti-DNA antibody may lead to an autoimmune disease is through its interaction with cell-death receptors, initiating a pro-apoptotic signal that apparently leads to tumor cell death in vitro and perhaps lupus B-cell death in vivo [12, 15, 26, 27]. Although the phenomenon is partially due to the activation of caspase, the possible link between DNA hydrolysis and programmed cell death, as well as underlying mechanisms in SLE, are not clearly understood [12]. The differences between natural and pathogenic anti-DNA autoantibodies are summarized in Figure 1.

**Figure 2:**
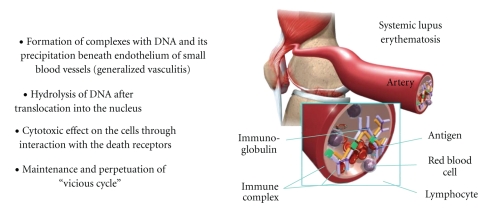
Lupus anti-DNA antibodies and their importance in SLE pathogenesis.

Beside their direct hydrolytic and cytotoxic effects upon the cells observed in vitro, the pathogenic mechanisms involved in SLE include the induction of initial lesions via deposition of circulating immune complexes (composed of lupus DNA and antibodies bound to it) into the tissues of various organs in vivo, thereby inducing an inflammatory response leading to multiple organ/tissue damage in the form of associating disorders such as systemic vasculitis, glomerulonephritis, chorea, and others. The correlation of these antibodies with SLE is undoubtedly established and summarized in Figure 2. The most important pathological finding in nephritogenic lupus is the presence of immune deposits beneath the glomerular endothelium in kidney biopsies and virus-like particles in endothelial cells [28]. The presence of virus-like particles in different organs in patients with lupus indicates that the anti-DNA antibodies could be a part of the body's viral or bacterial defense mechanism involved in the removal of foreign DNA during the course of the disease [29].

Anti-double-stranded DNA antibody is considered a hallmark of lupus disease, found in approximately 70%–90% of patients with SLE (especially those with nephritis), and measuring its levels in patient's plasma is used to follow the course of disease. However, since anti-single stranded DNA antibody could be both hydrolytic and nephritogenic, it may serve as a strong flare predictor in the course of the disease as well [4, 5]. The important role of anti-single stranded DNA antibody is supported by findings in mouse models of nephritogenic lupus in which only anti-ssDNA antibodies were found [30]. Furthermore some anti-dsDNA human antibodies are apparently not pathogenic l [7, 17]. Due to these controversies, a major goal in this field of research is to determine the optimal conditions for analysis of the types and activity of highly purified human anti-ssDNA (IgG and IgM) antibody, which could provide an insight into their role in SLE pathogenesis. Achievement of this goal could further develop means to produce highly purified anti-DNA antibodies to be used in numerous therapeutic studies. Highly purified antibodies may be useful in constructing dendritic cell-based anti-idiotype vaccines and idiotype peptides that may help regulate the disease [31–38]. Some differences between anti-ds and anti-ss DNA autoantibodies are shown in Table 1.

### 2.2. Isolation and Purification of Anti-DNA Antibodies

#### 2.2.1. Previous Attempts

Antibodies may play three important roles in auto-immunne diseases: protective, predictive, and pathogenic [40]. A major prerequisite for the study of their roles in maintaining health or eliciting disease is obtaining them in high purity. One fundamental objection to previous attempts at purifying anti-DNA antibodies directly from human plasma by Kozyr [26] is that the process yielded numerous bands on electrophoretic separation, and some bands were inconsistent with the molecular weights of IgGs. Furthermore, there was no attempt to separate or to distinguish between the antibodies against ssDNA and those against dsDNA, so the relative importance of the pathogenic role the two types play in SLE or their predictive role in the flare could not be determined [4, 5]. Therefore, the development of a specific method for isolation and purification of each of the two categories of anti-DNA antibodies with the highest level of purity detected using a highly sensitive system, is highly desirable. Such a method would allow parallel studies of the unique functional activities of the two types of antibodies as well as their specific role in the etiopathogenesis of SLE and other autoimmune diseases. Since antibodies may form complexes with other proteins, it is important to prove that the catalytic activity ascribed to anti-DNA antibodies is not due to a contamination with DNAse or some other hydrolytic protein but due to the fact that it is an intrinsic, constitutive property of the antibody alone.

The methods created and applied in the past were mainly done to detect the anti-DNA structural and functional characteristics (e.g., substrate binding, hydrolytic, and cytotoxic activity). A very reliable but laborious method was created by Puccetti et al.[41] In this attempt, the IgG fraction was bound to protein A Sepharose, eluted, and followed by native dsDNA cellulose in order to selectively capture anti-dsDNA antibodies. The bound fraction was thereafter eluted from dsDNA cellulose and dialyzed. The effluent was absorbed with poly-(dT) cellulose to bind anti-ssDNA antibodies and the final fraction was eluted with citrate buffer at a low pH. Thus, both anti-dsDNA and anti-ssDNA fractions were obtained and analyzed for their hydrolytic activity, either separately or together. A major disadvantage of this method is the loss of any IgG_3_ subclass antibodies since these do not bind to protein A. The hydrolytic anti-DNA antibodies were reported to be IgG_1_ and IgG_3_ classes. However, at the time of the study, this was the only method available to separate anti-ssDNA from anti-dsDNA after many laborious steps using relatively specific substrates. Most of the authors used DNA-cellulose purification based upon the technique pioneered by Moss et al. [3] wherein the DNA bound either anti-ssDNA or anti-dsDNA or both directly from sera. This method is relatively simple and gives a good yield (about 70% of anti-DNA antibody which maintains 100% binding and hydrolytic activity). Gololobov et al. [42, 43] and Rodkey et al. [30] used a modification of this approach to purify the mouse monoclonal anti-ssDNA antibody and its Fab fragment (obtained by papain hydrolysis) with excellent results with respect to their activity.

In [42] Gololobov et al. published a method consisting of two affinity chromatography steps followed by ion exchange and gel filtration chromatography for the purification of antibodies from human serum that maintained DNA-hydrolyzing activity. Antibody Fab fragments, obtained by papain hydrolysis of antibody purified 130-fold, were shown to catalyze plasmid DNA cleavage as determined by agarose-gel electrophoresis and Linear Dichroism techniques. The Fab fragment was shown to hydrolyze double stranded supercoiled plasmid DNA by Mg^2+^ -dependent single strand multiple nicking of the substrate. Such antibodies have also shown ability to bind, but not necessarily hydrolyze, single stranded regions which are undoubtedly present in stretched double stranded DNA [44]. This type of hydrolysis mechanism suggests that the Fab fragment could belong to anti-ssDNA antibody and could be used as a criterion for separating anti-DNA antibodies specific for ss- or dsDNA. Characterization of antibodies that react with both ss- and dsDNA will require further molecular analysis of their structural, binding, and hydrolytic properties. To purify DNA-hydrolyzing antibodies, the authors used protein A after a 50% saturated ammonium sulfate precipitation and dialysis of the isolated samples twice for 4 hours against 500 volume of Tris buffer, followed by MonoQ FPLC NaCl gradient, subsequent glutaraldehyde-modified DNA cellulose chromatography and Superose 12 gel filtration FPLC. Hence, the total IgG fraction with DNA-hydrolyzing activity was isolated; the method, however, failed to separate ds- from ss-DNA antibodies. So, this extremely laborious technique, lasting several days, was not highly selective with respect to the substrate binding and specificity. Furthermore, protein A does not bind IgG_3_, the subclass which is considered to be highly hydrolytic.

In 1996, Kozyr modified the previous procedure by using saturated ammonium sulfate for total IgG precipitation and Cibracon Blue 3GA for fast albumin removal, followed by ion exchange chromatography on DEAE-Sepharose FF, protein G-affinity chromatography for specific binding of the total IgG fraction, and selection of DNA–binding fraction on Sephacryl. After repeated dialysis, washing, and elution steps a total anti-DNA hydrolyzing fraction was eluted in citrate buffer, pH 2.6, and stored in PBS with 50% glycerol. This was a very time-consuming procedure resulting in many bands on electrophoresis other than anti-DNA antibodies (IgGs). The author suggested that multiple bands resulted from incomplete cleavage of S-S bonds by reducing agent. Separation of the antibody subunits during purification procedure can generate such electrophoretic patterns. In addition, enzymatic contamination of the purified antibody still remained a strong possibility, since anti-DNA antibodies are known to react with other proteins (laminin, fibronectin, alfa-actinin), phospholipids, and heparin sulfate.

Swanson et al. [17] reported the use of a protein-agarose matrix covered with ssDNA in order to isolate an anti-ssDNA antibody fraction of anti-DNA autoantibodies and checked the binding activity of the fraction through poly-(dT) coated ELISA plates (considering that poly-(dT) is an immunodominant epitope). All of the monoclonal antibodies identified in the ELISA screen recognized ssDNA, and several of them cross-reacted with dsDNA (although with variable affinity), confirming that anti-ssDNA antibodies do comprise the bulk of lupus anti-DNA antibodies, and can react with both ss- and dsDNA. The quality of this method however, is not conclusive, since no analytical data was shown. To our knowledge, it has not been utilized by other workers and therefore, the advantages or disadvantages of the method could not be evaluated.

In 1998, Kozyr et al. developed the first affinity-capture assay (new in that they used paramagnetic Dynabeads to purify anti-DNA antibodies) in order to prove the association of DNA-hydrolyzing activity with the antibody fraction. They incubated biotinylated mouse antihuman IgG, with SA- (Streptavidin-) coated paramagnetic beads, for 5 hours at 4°C degrees. The anti-DNA purification was performed on a Sepharose column, followed by addition of the mixture of antibodies to the beads in the ratio 2 : 1, and incubation for 1 hour. The SA coated beads which have bound biotinylated mouse antihuman IgG complexes on their surfaces will have human anti-DNA bound on the opposite end of the biotin chain. After washing the antibody mouse-human complex in the binding buffer and disrupting it with glycine-HCl (pH = 2.6), DNA abzymes were detached and free in the solution. The beads were removed by the magnetic separation device, the pH of the solution containing detached beads brought to pH 7.5 with 1 M Tris base and the final preparation checked for DNA hydrolyzing activity. This method is quite laborious, time-consuming, and utilizes harsh conditions to disrupt antibody complexes followed by long overnight dialysis treatments. The disruption event can adversely affect the binding and functional capacity of the antibody preparation. In order to preserve the binding and functional properties of antibody, gentler, faster means for purification have been sought.

### 2.3. A Novel Approach for Isolation and Purification of ssDNA Binding Anti-DNA Autoantibody

**Figure 3:**
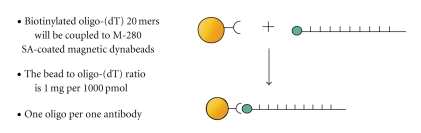
Oligo-dT beads preparation.

We have thus designed a novel two-step, efficient, fast, simple, and relatively inexpensive method for isolation and purification of anti-ssDNA antibody that yields a high level of purity [9, 10]. Our methodology for purification of anti-DNA antibody is based on the finding that anti-ssDNA antibody has a strong binding preference to thymine bases, confirmed by analysis of the crystal structure of a complex of oligo-(dT) and mouse monoclonal anti-ssDNA antibody [42, 45]. Tanner et al. in 2001 illustrated the specific binding of anti-DNA antibodies to thymidine polymers via arginine groups using X-ray analysis. The arginine groups are responsible for sequence recognition so that the antibodies bind the DNA at the thymidine repetitive sequences (5 mer) via tyrosine side chains within a hydrophobic pocket created by tyrosine and tryptophan from the antibody binding site. By using biotinylated oligo-(dT) 20 mer bound to streptavidin coated magnetic Dynabeads, it was possible to isolate anti-ssDNA antibodies specific for this single strand of thymine nucleotides [10]. Dynabeads M-280 Streptavidin coupled with Biotinylated oligo-(d) thymidine (20 mer) were used to isolate anti-DNA antibody directly from the patient's serum as the first step, followed by further purification of anti-DNA antibody using protein G Dynabeads (known to bind total IgG fraction) as a second step [9, 10]. The preparation of oligo-dT beads is schematically presented in Figure 3.

Due to methods of purification that in the past did not clearly distinguish anti-ssDNA from anti-dsDNA autoantibodies, it was unknown if the ELISA reaction is with the DNA in double stranded or single stranded configuration of the loops which usually exist within stretched dsDNA, as it was confirmed by the work of Mostoslavsky et al. [46]. We do know that the substrate for our anti-DNA antibody in our ELISA plates was single-stranded DNA specific (oligo-(dT)), and that it reacted exclusively with ssDNA-coated ELISA plates. Obviously dsDNA may contain sequences of repeated thymidine to which the apparently single strand-specific antibody could bind. 

However, Oligo-T-purified anti-ssDNA-IgG was of extreme purity as evidenced by the appearance of a single band following very sensitive silver-staining scale utilizing the Pharmacia PhastGel electrophoretic staining method (GE Healthcare, Piscataway, NJ; Owners Manual Separation Technique File No.130), where there is between 0.3–0.5 ng of protein per band, and within the range of the sensitivity of silver method applied, between 0.1–100 ng of protein [46]. There may however be traces of other proteins or antibody classes whose concentration falls below the level of detection by silver staining.

The characteristics of Dynabeads make them suitable for molecular purification. Dynabeads M-280 Streptavidin coupled with biotinylated oligo-(d) thymidine have an excellent stability and a high lot-to-lot reproducibility due to the low nonspecific binding characteristic of streptavidin and high binding affinity of the streptavidin/biotin interaction (KD = 10–15) which allows for efficient isolation of the whole (intact) target molecules (no separation of subunits in nonreducing conditions). The beads were blocked during manufacturing procedure (personal report from the manufacturer) with 3% BSA to eliminate nonspecific binding for SA and after that, kept in the storage buffer with 0.1% BSA. It is known that some anti-DNA antibodies to Streptomyces avidii (a rare cause of chronic respiratory infections in humans) can bind to only SA-coated beads. Therefore, it can be bound in the clefts between biotin molecules (since 1 SA molecule binds a total of 4 biotin molecules on the entire surface of 4 SA subunits-one molecule of biotin per one subunit of SA). Their interference in our system is excluded due to the previous blockage of the beads which are subsequently checked for the binding of purified antibody to beads coated with SA alone. A dramatic drop was found in the yield of anti-DNA antibody with recycled beads. 

Protein G Dynabeads are designed for IgG isotype purification. Protein G has a strong binding affinity to four different isotypes of IgG, and there is no leakage of protein G from the bead surface beads during purification. Moreover, Dynabeads freely suspended in a solution can be washed many times to eliminate nonspecific binding. Another advantage of this method is that we can calculate the optimal number of the beads required for the amount of antibody determined by immunoassay. The oligo-(dT) coupled to the SA Dynabeads is a 20-mer poly T which has the feature of DNA, but is too short to bridge two antibody molecules [17]. Because of the characteristic of oligo-(dT) 20 mer, we are able to estimate the maximal amount of the beads used to recover all anti-DNA antibodies from 1ml of patient's plasma. This makes the method very precise and economical for application. 

In summary, previously reported methods for anti-DNA antibody isolation and purification involved a combination of biochemical and affinity matrix steps, many of which are nonspecific. Moreover, the abzyme (named according to Linus Pauling, 1946) characteristic of anti-DNA antibody, prone to denaturation under such harsh conditions, hinders the routine use of these methods. Thus, nonspecific binding and denaturation have been major issues regarding the purification of anti-DNA antibody. In order to explore the structure, function and properties of anti-DNA antibodies, many scientists shifted their attention to monoclonal anti-DNA antibody generation. Mouse monoclonal antiss-DNA antibody of the IgG isotype (IgG_1 _and IgG_3 _specifically) has a high affinity to antigen and has been shown to be hydrolytic [30]. Although hybridoma technology is easily applied for studies of autoantibody in mice, it is limited for human autoantibody generation. The major problems include immunization, the source of immune lymphocyte, the limitation of the immortal fusion partner, and the unstability of the hybridomas. More importantly, the monoclonal antibody does not represent the spectrum of anti-DNA antibody subclasses present in a patient's serum. To date no information is present regarding the subclasses exhibited in diseased versus normal human individuals. For that reason, the development of a simple, specific and moderate method of purifying anti-DNA antibodies from patient's serum or plasma, such as the 6-hr procedure with magnetic beads, is important for the study of autoimmune disease.

To our knowledge, our lab was the first to develop a specific and simple method for isolating and purifying human anti-DNA (IgG) antibodies from the SLE patients' serum, based on the specific binding of anti-DNA antibodies to thymine polymers (anti-DNA antibody base specificity is dT ≫ dG ≫ dC ≥ dA) via arginine groups within the antibody [3, 17, 45]. Based upon this finding, it was possible to isolate anti-ssDNA antibodies specific for a single strand of thymine nucleotides by using biotinylated oligo-(dT) 20 mer bound to streptavidin (SA)-coated magnetic Dynabeads from Invitrogen (Carlsbad, CA) [10]. Our attempts to purify anti-ssDNA antibodies using other oligomers (dC, dG, and dA) bound to streptavidin-(SA-) coated Dynabeads were unsuccessful. 

**Figure 4:**
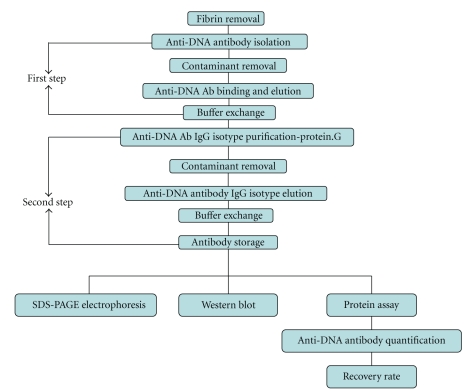
Scheme of two step magnetic bead based purification of IgG from serum of patients with SLE.

**Figure 5:**
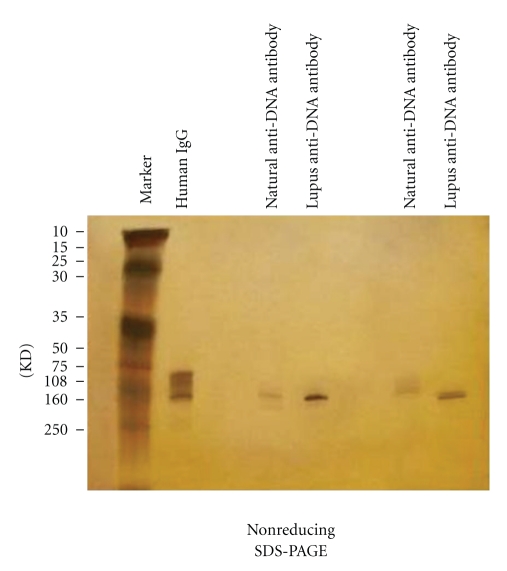
Electrophoretic analysis of the purity of anti-DNA antibody purified via two-step affinity method employing magnetic beads.

 The principle of two-step magnetic bead method for purification of anti-ssDNA is presented in Figure 4 and the results of purity on SDS-PAGE in Figure 5.

## 3. Idiotypes and the Role of Idiotypic Network

Idiotypes are the antigenic determinants of immunoglobulin molecules that are located in the variable region of the antibodies. Idiotypes are subdivided into those that reside at the antigen-binding site, the paratope of the antibody molecule, and those on the areas adjacent to this site, the framework determinants. The potential role of idiotype-anti-idiotype interreactions in the immune system stimulated Jerne to postulate the presence of an “idiotypic network” through which immunoglobulin expression might be controlled [31]. Idiotypic dysregulation is now considered one of the major mechanisms that may underlie antibody-mediated autoimmune disease. 

According to Shoenfeld and Mozes, experimental SLE could be induced in mice by anti-DNA idiotype immunization and could be abrogated by anti-idiotypic and intravenous immunoglobulin treatment [47]. However, there are also reports that idiotypic network can be used for the production of catalytic autoantibodies [33]. 

## 4. Detection Methods

As already mentioned, the presence of antibodies against double-stranded DNA (dsDNA) is considered to be a hallmark of Systemic Lupus Erythematosus (SLE) and thus is used as one of the diagnostic criteria for the disease. Recent research has shown that antibodies against single-stranded DNA (ssDNA) may also play a critical role in the course of the disease and in disease pathogenesis. However, currently, physicians continue to focus only on monitoring levels of anti-double stranded DNA antibodies in the blood of patients diagnosed with lupus.

There are essentially three techniques used to measure levels of anti-DNA antibodies in the blood:

- Crithidia lucilliae immunofluorescence assay,
- ELISA techniques,
- Farr assay (radioactive).

The quantitative determination of anti-DNA autoantibodies (particularly dsDNA) is useful for monitoring patients, particularly those with symptoms of nephritis. For monitoring in these clinical cases, a quantitative assay is recommended (Farr or ELISA-[48]).

### 4.1. Crithidium lucillae Immunofluorescence Assay

Anti-dsDNA autoantibodies are highly specific for SLE and are present in a high proportion of SLE patients 40%–80% [48]. Another method employs the microorganism Crithidia lucillae for diagnostic purposes because of its high specificity.

Authors/Commercially Available Kits. Zuess Scientific Inc, Rarian, NJ, USA Commercially Available Kit., 2002). As an alternative, Crithidia lucillae can be used in diagnostic purposes for its high specificity.

**Principle**

Indirect immunofluorescence assay—dsDNA from the microorganism Crithidia lucilliae is used as the substrate for anti-DNA antibody binding, and patient sera diluted 1 : 10 are added to each well. After incubation, the reaction is washed 3 times, and antigen-antibody reaction is determined by FITC-labeled antihuman immunoglobulin. An epifluorescence microscope from Zeiss-Axiophot is used to read the slides. A positive test is considered at a titer of 1 : 10 or above.

**Range of Sensitivity**

Qualitative—low sensitivity.

**Cost**

Average is $8.

**Advantages**

Simple, inexpensive, reliable, and more extensively in use by the majority of clinical laboratories. The main advantage of the assay is the presence of a highly stable dsDNA that is concentrated in the kinetoplast (structure involved in movement of the organism), thus giving very high specificity of anti-dsDNA detection. The specificity of the reaction is high and there is no use of radioisotopes. An additional advantage is that the test requires little technical expertise.

**Disadvantages**

Subjective, semiquantitative, and relatively low sensitivity. Also, false positives are found sometimes due the presence of antihistone antibodies (histones are proteins that bind DNA). There is kit-to-kit variability due to the preparation of the Crithidia lucilliae substrate.

### 4.2. Commercial Kits Using Different ds- and ss-DNA as a Substrate (ELISA)

It is not advisable to use ELISA for diagnostic purposes due to its low specificity. The affinity-linked oligonucleotide nuclease assay (ALONA) was introduced by Mouratou et al. [49]. This test uses digoxigenin coupled to the 5′ end of a 3′-biotinylated DNA strand. In order to detect catalytic anti-DNA antibodies which release the digoxigenin from the substrate. This test is considered to be somewhat more specific than ELISA itself, but still not as specific as the Farr's assay. In determining the level of anti-ssDNA, most of the authors agree that either bacteriophage or plasmid DNA should be used as a substrate rather than calf thymus or any mammalian DNA. This preference is mainly due to the long and fragile nature of mammalian DNA which usually unwinds partially and binds ss-DNA antibodies in the test system for anti-dsDNA determination, creating false negative results [5, 44].

Authors/Commercially Available Kits. Kits from Diamedix Corporation, Miami, FL, USA; SCIMEDX Corporation (for ds and ss DNA); ANTI-ssDNA Test from AtlasLink; Diagnostic Automation Inc.; MESCUP DNA-II TEST “ss” (for ssDNA); Helix Diagnostics, (dsDNA)West Sacramento, CA.

**Principle**

ELISA test; 96-well Micotiter plates are coated with highly purified calf thymus ds or ss DNA. In each well usually 100 microliters of diluted patient sera is added; after incubation, each plate is washed 3 times. In addition 100 microliters alkaline phosphatase conjugated anti-human IgG is added. After incubation and washing, the substrate is added and the color development is read at 405 nanometers. The results are expressed in international units per mL by using a single point calibrator provided in the kit. A negative value was considered as less than 100 international units per mL. A value of 1–300 IU/mL is considered borderline, and greater than 300 is considered positive.

**Range of Sensitivity**

>300 IU/mL.

**Cost**

$4.80, when done single and $9.00, when done in duplicate.

**Advantages**

Good screening test for SLE; Immunoglobulin class-specific antibody detection; No problem of isotope disposal (no radioactivity); technically easier to perform than Farr.

**Disadvantages**

One of the most important problems with the ELISA assay is determining what cutoff level should be used when interpreting the results. A second important point is the measurement and clinical significance of low avidity and/or IgM anti-dsDNA antibody of ELISA assay. Due to great variations between different kits and between samples of the same concentration within the same kit, it cannot be considered highly reliable. It is advisable to confirm the result of the ELISA with a more reliable assay like the Farr Assay (RIA→ radioimmunoassay). Takes 4–6 hours to perform.

### 4.3. Farr Assay-Radioactive

This is the defining assay for detecting high avidity anti-dsDNA. It is also the method of choice for detection of antibody to native double-stranded DNA but should not be used as a screening test to detect SLE [48]). This test is usually performed on patients already known to have SLE, but is also performed on patients who are screened for ANA (antinuclear antibodies) and are reactive (have the symptoms of lupus). The necessity of using radioactive material decreases its applicability. 

Authors/Commercially Available Kits. Diagnostic Products Corporation, Los Angeles, CA, USA.

**Principle**

I-125 labeled recombinant DNA (very pure) is incubated with 25 microliters of patient sera. After incubation, bound antibody is carefully separated from unbound antibody using ammonium sulfate, and the bound fraction is counted by a gamma counter. The anti-dsDNA antibody level is determined by using a standard curve prepared from the previously calibrated standard curve based on WHO First International WO/80 standard, which is provided in the kit.

**Range of Sensitivity**

>4 IU/mL.

**Cost**

$5.60, when done singly and $10.00, when done in duplicate.

**Advantages**

High sensitivity due to radioactive (iodine) labeling of the substrate (DNA) takes about 3 hours to perform highly pure recombinant human DNA and highly pure bound fraction (precipitated by ammonium sulfate).

**Disadvantages**

Use of radioactive materials which require safe handling and disposal no differentiation of isotypes of the dsDNA antibodies; the test does not detect antibodies of low avidity and/or low affinity anti-ds antibody. Relatively high level of technical expertise is required to perform the test. A dilution series of sera giving >50 IU/mL for exact quantitation is necessary. Shelf-life of the kit is only 6 weeks.

## 5. Recognition, Binding, and Functional Activities

What is the exact role of DNA-reactive antibodies in lupus patients? What part of the abzyme possesses catalytic activity? Fab fragment? Or could a single heavy or light chain possess this activity? What is the exact role of these antibodies in lupus? What part of their molecule possesses catalytic activity? Fab fragment? Or either heavy or light chain? Does the Fc fragment inhibit or interfere with the enzymatic activity of the molecule as proposed by others? [30, 43]. The later observation is particularly noteworthy as it may afford a means to regulate the activity of these unique abzymes. It is somewhat puzzling that healthy individuals as well as SLE patients produce anti-DNA antibodies which can be isolated from their seruam [9, 10, 15, 50]. However, normal individuals do not appear to be adversely affected by their presence, and their anti-DNA antibodies are not hydrolytically active. Hydrolytic activity has only been seen in anti-DNA antibodies produced in the disease state [15, 50]. Why are they then present in normal or apparently healthy individuals? Catalytically active antibodies are found in the sera and milk of pregnant and lactating women and are considered to provide a maternal strategy to protect against microbial attack of the fetus and newborns [10, 50]. In addition to anti-DNA antibodies, anti-RNA, NMP, NDP, and NTP as well as antibodies with proteolytic activity have been found in human sera of healthy and diseased people, suggesting that the human host is trying to fight a microbial agent. Microbial DNA (bacterial and viral) is a known immunogen and some authors suggest that a high frequency of unmethylated CpG motifs in microbial DNA acts as a stimulator of anti-DNA antibody production and the flare of symptoms seen in lupus. The CpG motifs act through binding to toll-like receptor 9 (TLR-9) on B cells and plasmacytoid dendritic cells [10, 51, 52]. 

Studies show that anti-DNA antibody binds to peptide-mimicking antigens which were synthetically designed, some of which mimic viral or bacterial proteins [53]. One of the candidates for the appearance of anti-ssDNA antibody could be the only known human ssDNA virus, parvovirus B19 which has all possible characteristics [54] of a causative/triggering agent (in at least a small fraction) of lupus patients. Parvovirus can be found in most humans as it resides in the erythroid precursor in a latent state; however, symptoms only appear in a few patients. However, when symptoms are displayed, they closely mimic lupus symptoms. The spectrum of lupus symptoms and the heterogeneous nature of the disease suggest that it is probably caused and triggered by multiple environmental factors (similar to cancer) and therefore, requires individual diagnosis, therapy, and prevention [18, 53, 55–57]. Perhaps prevention of a fraction of lupus patients whose disease is caused by parvoviruses is possible by early childhood vaccination against the virus [10].

## 6. Mechanisms of Action

### 6.1. Recognition and Binding

Antibodies to DNA serve as models for the study of protein-DNA recognition. For these studies, monoclonal antibodies produced from hybridomas were used. In many cases it is not possible to produce sufficient Ig, mostly for these studies. Human monoclonal antibodies, especially of the IgG isotype, were particularly difficult to obtain until the method of repertoire cloning was developed [58, 59]. This approach enables the generation of monoclonal antibodies of defined specificity through the molecular cloning of expressed Ab heavy (H) and light (L) chains genes that are isolated directly from lymphocytes. The genes are selected, based on their binding specificity after in vitro expression [59]. An alternative approach is to use computer-modeling techniques to examine the antigen-combining sites of anti-DNA antibodies [60]. It has been shown that anti-dsDNA autoantbodies prefer phosphate backbone for binding [61–64] while anti-ss-DNA prefer oligo-dT [17, 45]. Anti dsDNA also react with planted antigen [17] and bind to cellular proteins [60, 65, 66]. On the basis of experiments with monoclonal antibodies, it was suggested that anti-dsDNA monoclonal autoantibodies exhibited preference for DNA-binding motifs different than those of anti-ssDNA autoantibodies. Wang et al. [60] have shown that the fragments of ssDNA or dsDNA form bound by the same stock of antibodies were different in their conserved sequences. Furthermore, ss-DNA fragments recognized by anti-DNA antibodies were rich in the following nucleotide sequences:

**-cacc**

**-caccg**

**-accc**

**-cccc** blocks, while the same stocks of antibodies exhibited significant preference for −5′ gcg 3′/3′cgc5′ motifs located in dsDNA [60]. According to Swanson's binding studies [17], both dsDNA and ss-DNA can deposit in patient tissues and glomeruli via a process involving DNA binding. Anti-ss-DNA Fab fragment denoted DNA-1 was isolated from a combinatorial bacteriophage display library of IgG fragments derived from the immunoglobulin repertoire of an autoimmune SLE MRL/lpr mouse. This fragment binds to oligo-(dT) 15 nucleotides or greater in length with a Kd of 140 nM. The Kd values for poly dG, dC, and dA being of 1–10 uM indicated its marked preference for oligo (dT). Furthermore, Tanner et al. [45] revealed the crystal structure of monoclonal mouse anti-ssDNA autoantibody, for example, a crystal structure of recombinant Fab (DNA-1) in complex with dT5 (Thymidine pentamer). The available crystal structures indicate that DNA binding causes significant conformational changes in the antibody, probably leading to catalytic activity. It is widely believed that high arginine content is a fundamental property of the hypervariable loops of both anti-dsDNA and anti-ss-DNA antibodies from lupus prone mice and SLE patients. Indeed, the affinity of autoimmune antibodies for dsDNA can be increased by introducing Arg into the H3 domain, while elimination of Arginine has been shown to reduce the affinity for dsDNA. Likewise, Arg in H3 is thought to be critical for binding ss-DNA. Crystal studies of Tanner et al. [45] suggests that in the case of mouse monoclonal antibody BV-04 Arginine within the antibody is responsible for recognition while Tyrosine is needed for the binding to the sliding thymidine pentamer. For some reason the pentamer is necessary to attract the antibody but only T1, T3, and T4 are involved in binding [45]. At present, no crystal structure of an anti-dsDNA antibody that is complexed with its antigen is available. Although different mechanisms are used for binding ss and ds ligands, the mode of DNA recognition appears to be conserved within groups of antibodies [17] (Table 2).

The pathogenic anti-DNA antibodies might have dual activity: hydrolysis and cytotoxicity, the latter at least expressed toward certain tumor cell lines [13, 14].

### 6.2. Hydrolysis: Mechanism of Action

**Figure 6:**
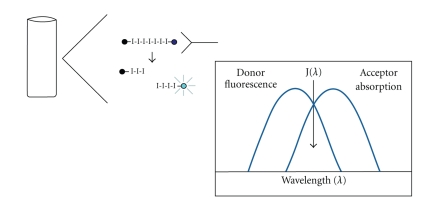
Continuous fluorescence-based hydrolysis assay.

**Figure 7:**
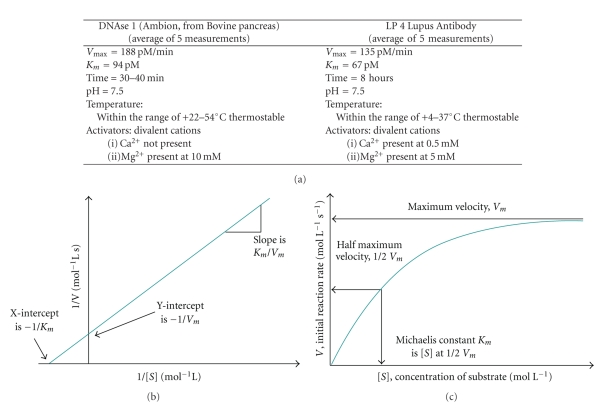
Kinetic parameters of DNAse 1 and lupus anti-ssDNA antibody.

The prerequisite for binding of anti-DNA antibodies is the recognition of either a phosphosugar backbone of dsDNA (for anti dsDNA autoantibodies) or a thymidine pentamer in ssDNA (for antiss-DNA autoantibodies) while the prerequisite for hydrolysis is binding itself. In der to hydrlolyze DNA, antibody must be tightly bound to it. According to Kozyr [14], only a certain subset of anti-DNA autoantibodies reveals the features of DNA hydrolysis (70%) and tumor cell line cytotoxicity (30%), thus outlining the presence of specific functionally active structures. For at least one mouse monoclonal anti-ss-DNA autoantibody (BV04-01), the active center is known to be HisL27, which intercalates between the second and third thymidine residues in ss-DNA. This anti-ssDNA autoantibody has been proven to have hydrolytic activity in the presence of Mg^2+^ and Ca^2+^ with Mg^2+^ acting at the center of the active site, while Ca^2+^ accelerates hydrolysis. Catalysis was associated with Fab and single-chain antibody proteins [30]. According to the same author, the percentage of DNA-hydrolyzing antibodies in SLE patients was 42%, while in B-cell tumors it was 52%. Very recently, the studies of Parkhomenko et al. [67] have shown that hydrolytic activity is localized in the light chains of the antibody molecule. The other details of the process are unknown. Further research is necessary to reveal the specificity of antibodies for nucleotide sequences and kinetics of hydrolysis. The initial data, based on the novel real-time fluorescent assay [68] **(** Figure 6 **),** suggest that the reaction differs fundamentally from isolated DNAse I activity, indicating that it is an intrinsic property of the antibody itself [69] **(** Figure 7 **)**. Are the cytotoxic and hydrolytic active sites of the abzyme seen in lupus patients located in the same or different parts of the molecule?

### 6.3. Cytotoxicity: Mechanism of Action

Only 30% of anti-DNA autoantibodies isolated from the patients with either CLL or SLE revealed antibody cytotoxicity to tumor cell lines [27]. In only one case of each disease did DNA specific antibodies exert catalytic activity that did not accompany cytotoxicity, suggesting different active sites in these exceptions. It seems that there may be several mechanisms of cytotoxicity induced by these antibodies. Cytotoxicity is detected at an antibody concentration of 10^−8^–10^−10^ M, which is comparable to the cytotoxicity of TNF-*α*. Time-dependent profile of anti-DNA antibody cytotoxicity has revealed two distinct peaks by 3 and 18–48 hours when L929 cells were incubated with 10 nmols of anti-DNA antibodies. [13] Suchkov, et al., (2001) in trying to explain the underlying mechanisms of cytotoxicity, suggested that several apoptotic mechanisms could be involved considering initial activation of caspase at the early stages of the anti-DNA antibody action on the cells. Kozyr et al. [14] suggested that the mechanism of interaction of the antibodies within the cells might be cell cycle-dependent. These data may outline the pathogenic role of DNA-hydrolyzing autoantibodies upon entering the nucleus, thus contacting with chromatin (cross reaction with nuclear matrix proteins) and causing DNA cleavage through induction of apoptotic pathways, with consecutive cell death. Anti-DNA antibody-mediated cell death may be attributed to activation of signal transduction pathways and apoptosis. Alternatively, anti-DNA antibodies may enter the cell and either block protein synthesis or, in case of DNA hydrolyzing antibodies, cleave DNA. Part of the pathogenicity of anti-DNA autoantibodies might be due to cell destruction, leading to the release of nuclear contents and thus new antigens into the blood stream with perpetuation and maintenance of the anti-DNA autoantibody production. The functional properties of anti-dsDNA and anti ss-DNA antibodies are summarized in Table 3.

## 7. Pathogenicity

There is no general consensus regarding the mechanism of pathogenicity and possible differences in the pathogenicity between anti-dsDNA and anti-ss-DNA autoantibodies in SLE. Some authors consider anti-dsDNA to be pathogenic, while anti-ss-DNA is nonpathogenic. Those who consider anti-dsDNA autoantibody to be pathogenic describe it as a high affinity cationic IgG molecule (preferentially of IgG3 isotype) that binds dsDNA and fixes complement. These complement activating immune complexes are thus considered the cause of general vasculitis and especially, glomerulonephritis [55]. The variable region of autoantibody exhibits somatic mutations and is enriched with acid residues that are appropriate for DNA binding. The presence of Arg on DC-R-H3 is particularly notable. However, Ehrenstein et al. [70] suggested that anti-dsDNA is not pathogenic at all. Swanson [17] has shown that some monoclonal anti-dsDNA antibodies are pathogenic, while some of them are benign. In the same experiment he showed that anti-ss-DNA can be even more pathogenic than anti-dsDNA, as judged by the binding activity. The later results are supported by the work of Sun et al. [71]. Aotsuka and Yokohori [4] have found that anti-ssDNA antibodies of IgG class showed a significantly higher value in sera compared to other isotypes and correlated well with lupus urinary abnormality. He also noticed that there were the patients with SLE in an active disease who presented with increased levels of anti-ss-DNA antibody (IgG class) just prior to the disease period (reported in MESACUP DNA TEST “ss” medical and Biological Laboratories, Inc). Teodorescu [5] performed a longitudinal clinical study in a group of lupus patients and concluded that levels of anti-ss-DNA autoantibodies are the best predictors of a forthcoming increase in dsDNA levels and the SLE symptom flare. Beckingham et al. [6] considers anti-ss-DNA pathogenic because of its sequence recognition of ssDNA and binding and pointed to the strategies for disrupting DNA binding that could prove to be therapeutically useful. Quite recently, Chen [9], Pavlovic et al. [10, 72] used self-created magnetic oligo-dT beads for the isolation of a highly pure human lupus anti-ss DNA antibodies. The later have shown hydrolytic activity upon a ssDNA probe in a sophisticated newly designed [68] fluorescent real-time assay system and related the enzyme activity to the pathogenic role of these antibodies. What is the physical basis for the differential pathogenicity? What makes some anti dsDNA autoantibodies clearly more pathogenic than others? Kalsi et al. [18] suggested that pathogenicity might not be related to the charge and affinity of anti-DNA autoantibody, but to its fine binding specificity. Perhaps B cells responding to the flare in replication of a single stranded viral agent, like parvovirus, initially produces a wave of anti-ssDNA antibodies. As the immune response continues in an effort to control the agent, affinity maturation and epitope spreading within the B cell's progeny may lead to the production of anti-dsDNA antibodies. If the single stranded viral agent utilizes double stranded replicative intermediates, the necessity of producing hydrolytic antibodies becomes evident. Should the viral agent reside within the nucleus, perhaps cytotoxic antibodies play a key role in host defense against these agents. However, the role of anti-DNA antibodies in humoral defense against viral agents has not been investigated and remains an untapped field of investigation.

## 8. Treatment

### 8.1. Plaquenil

**Figure 8:**
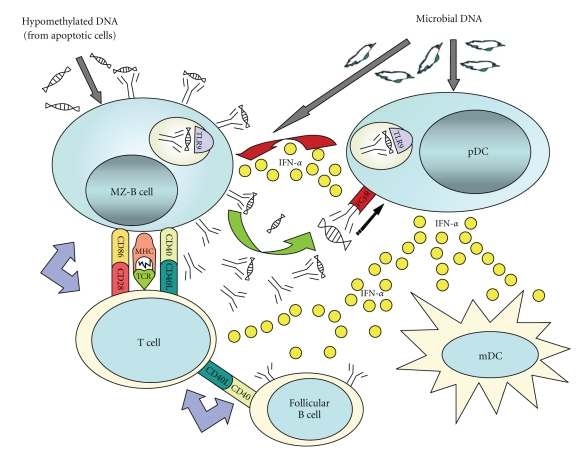
The innate model of lupus pathogenesis.

These are antimalarial drugs, particularly effective for joint pain, rashes, and mouth ulcers (about 50% patient's response). They are very safe, except for some concerns regarding retinal toxicity. The drug is sometimes recommended to pregnant women as there have been no cases of defects in the newborn, and there is a risk of a flare in disease symptoms in the mother if the drug is discontinued. The mechanism of action of these drugs is not known. However, recently, it has been shown that palquenil acts through TLR9 by decreasing the production of anti-DNA autoantibodies in patients with rheumatoid arthritis [51, 73]. This fits into the “Theory of innate immunity“ recently applied to SLE (Figure 8) [51] and lends credence to the role of microbial DNA in eliciting the disease. 

### 8.2. B-Cell Depletion Therapy

Rituximab is a chimeric (human/mouse) monoclonal antibody that is specific for the B-cell surface marker CD20 and aims towards B cell depletion. It is currently used to treat leukemias and lymphomas. Recent studies and clinical trials have shown successful results in SLE treatment [74]. Initially, investigators theorized that by removing the anti-DNA producing B-cells, they would be replaced by new nonself reactive B cells that would produce protective antibodies. The clinical trials involving patients with rheumatoid arthritis and lupus are ongoing. In the Medizinische Klinik, Univeristatsklinikum in Freiburg, Germany, treatment with Rituximab successfully and dramatically improved a 59-year-old women's life, which was unresponsive to the typical SLE medications such as methotrexate, corticosteroids, and azathioprin. This patient went into complete remission 18 months after the treatment was completed. The results from other studies [75–77] indicate that while patients are under the treatment the anti-DNA antibodies disappear leading to remission of symptoms. Eventually, the flare may reoccur in some patients and therefore, this kind of therapy is not causal as it was curative as expected. Deeper knowledge at the causative level of SLE is necessary in order to effectively cure the disease. In terms of therapeutical approach, it is worth to mention the work of [35, 57, 78] on anti-DNA antibody. Idiotypic vaccines developed anti-DNA antibody vaccines in mice. The approach was to load dendritic cells with anti-DNA idiotype in order to elicit cytotoxic T cells which would destroy the lupus B cell. This approach is in developmental stage and requires more basic knowledge of immunology.

## 9. Induction and Regulation of Anti-DNA Antibody Responses by Environmental Factors

### 9.1. Phthalate, Pristane, and Vaccines

Environmental substances are considered important triggers of the disease state in lupus patients. They entail infectious agents, chemicals, drugs and even vaccines. Little is known of the role of specific environmental factors in promoting autoimmune disorders such as systemic lupus erythematosus (SLE). Lim and Ghosh in 2005 conducted a study on how exposure to phthalates, common environmental factors in foods, and biomedical devices could affect the immune functions of resistant and autoimmune-prone mice. They have previously shown [79] that immunization with orthophthalate evokes anti-DNA antibody in BALB/c and NZB/W F1 mice, but only the latter suffered from nephritis and high mortality. BALB/c mice, in contrast, developed idiotype-specific CD8+ suppressor T cells (Ts) that downregulated autoreactive B cells. In their following study they reported that all phthalate isomers (ortho-, meta- and para-) are capable of inducing anti-DNA antibody responses and SLE-like syndromes. Kidney pathology worsens in NZB/W F1 and to a degree, in C57BL/6 mice after repeated exposure to phthalates [80]. Only BALB/c and DBA/2 overcome adverse autoreactivity by induction of Ts cells, but in vivo depletion of these T cells renders these strains susceptible to autoreactivity. Anti-DNA antibodies in affected NZB/W F1 are largely IgG2a-type, while in BALB/c, DBA/2, and C57BL/6 mice IgG1-type. This was further corroborated by cytokine analyses that imply corresponding Th1/Th2 involvement. In summary, the commonly used phthalates appear harmful to susceptible strains, while BALB/c and DBA/2 are spared due to induction of Ts cells. Ts cells have a known effect of suppressing polyclonal B-cell expansion, the event which consecutively leads to lower anti-DNA autoantibody secretion. It would be revealing to determine if any human lupus patient has antibodies reactive toward isomers of pthalates and if healthy individuals possess T suppressor cells for this common environmental contaminant. Other environmental factors affecting anti-DNA autoantibody production involve pristane [81], vaccination with minigenes [82] or pConsensus peptide [83]. These agents possess inducing mechanisms similar to that of phthalates, with B-cell ablation by Ts cells again seen in nonsusceptible strains of mice.

### 9.2. Drugs

We have already described the drugs which are the most effective for anti-DNA antibody decrease and temporary disappearance. There is also a spectrum of drugs which cause so-called “drug-induced lupus”, but interestingly, these patients do not present with detectable anti-DNA autoantibodies. Therefore, in drug-induced SLE there is no nephritogenic syndrome and all signs and symptoms usually subside when the critical medication is no longer administered.

### 9.3. Infectious Agents and Production of Anti-DNA Autoantibodies

#### 9.3.1. State of the Art: Innate Immunity and Possible Conditions for Production of Anti-DNA Antibodies in Autoimmune Diseases

Recent studies have shown that infectious agents such as viruses, bacteria, parasites, fungi, and other organisms have variable effects on autoimmune disease, can induce autoimmune disease, enhance autoimmune disease; or even abrogate or offer protection from autoimmune diseases [46, 55, 84–90]. Increasing evidence shows that our immune system's normal response to infection results in an amelioration of autoimmune disease symptoms and protection against autoimmune disease development. This evidence supports the theory known as the hygiene hypothesis, which states that increased use of antibiotics, antibacterial and antiseptic cleaning agents, and vaccines leads to an enhanced incidence of autoimmune disorders, asthma, and allergies [84].

#### 9.3.2. Infection and Protection

Normally, infection of target cells and organs causes the release of sequestered (normally hidden from the blood circulation) autoantigens (self-protein particles) [84, 85]. Antigen presentation to immune system cells is thereby enhanced. Although these antigens normally provoke a heightened inflammatory response, some of them can activate regulatory T-lymphocyte cells, which dampen rather than evoke aggressive immune responses. Cytokines and chemokines released during the immune system's response to infection can direct aggressive T lymphocytes to the site of infection, drawing them from the autoimmune process. The autoimmune process that orchestrates production of anti-DNA autoantibodies seems to involve both innate and acquired immunity including Toll-Like Receptors, Inflammasomes, DNA-sensing proteins and transcription factors. The details of mechanism of secretion are still not known, but within last few years the emerging knowledge indicates that liaisons of the pathway(s) are becoming clearer.

**Toll-Like Receptors (TLRs)**

TLRs are arguably the most studied of the pattern recognition receptors (PRRs) [89–92]. They are transmembrane receptors that recognize individual pathogen-associated molecular patterns (PAMPs) on invading microbes. The intracellular tail contains a highly conserved region, called the Toll-interleukin-1 receptor (TIR) domain, which mediates interactions between TLRs and downstream signaling molecules [91].

Recognition of PAMPs by the TLRs triggers a series of events leading to the expression of many immune and inflammatory genes. TLRs also induce dendritic cell (DC) maturation, which is essential for the induction of pathogen-specific adaptive immune responses. One of the important lupus signature is the secretion of Interferon *α* by plasmocytoid dendritic cells ( pDC)(92). To date, 10 TLRs (TLR1-10) in human and 12 TLRs (TLR1-9 and TLR11-13) in mice have been described [92].

TLRs are expressed on a range of immune cells, which include macrophages, DC, B cells, and certain types of T cells [90]. They are also expressed on certain nonimmune cells, such as epithelial cells, which lie at potential sites of entry, including the skin, respiratory, intestinal and genitourinary tracts, endothelial cells, and smooth muscle cells. Their pattern of expression is modulated by activation, maturation or differentiation of the different cell types. TLRs 3, 7, 8, and 9 are expressed intracellularly, while TLRs 1, 2, 4, 5, 6, and 10 are expressed on the cell surface [89]. Briefly, TLR2, which works with TLR1 or TLR6, recognizes bacterial components, such as lipopeptide and lipoprotein of gram-positive bacteria; specifically, the heterodimer TLR1/2 which can recognize triacyl lipopeptide, while TLR2/6 recognizes diacyl lipopeptide.TLR7 is documented to recognize RNA, while TLR9-DNA, both microbial and self [89, 90].

**Inflammasomes**

Inflammasome is a name given to a large, signal-induced multiprotein complex that mediates the activation of proinflammatory caspases [93–95]. As a multiprotein complex it mediates the release of interleukin-1*β* from cells in response to infection by various pathogens or other danger signals. Interleukin-1*β* is a potent proinflammatory cytokine released from cells upon infection which triggers as well as directs the innate and adaptive immune response.

Quite recently, Muruve et al. [94] have reported that the inflammasome recognizes potentially dangerous cytoplasmic microbial or viral DNA and triggers an innate immune response [94]. Endosomal Toll-like receptor 9 (TLR9) and the cytoplasmic sensor DNA-dependent Activator of IFN-regulatory factors (DAI, also known as ZBP1) have previously been identified as intracellular receptors for DNA that trigger a type I interferon (IFN) response. Now, Muruve et al. report that activation of the inflammasome is also important for the generation of an effective inflammatory response to adenoviruses and other DNA viruses [94]. For example, infection of the human monocyte cell line THP-1 with adenovirus or herpesvirus led to pro-interleukin-1*β* (IL-1*β*) maturation and caspase-1 activation, which are both indicators of inflammasome activation. These effects were absent following treatment with empty viral capsids or extracellular adenoviral DNA, indicating that neither virion internalization alone nor extracellular DNA could trigger the inflammasome [93, 95].

A closer examination of inflammasome components revealed that the cytoplasmic receptor NALP3 (NATCHT-, leucine-rich-repeat- and pyrin-domain-containing protein 3; also known as NLRP3 or cryopyrin) and its adaptor protein, ASC (apoptosis-associated speck-like protein containing a caspase recruitment domain-CARD), are essential for sensing viral DNA, as macrophages from mice deficient in either of these proteins had significantly reduced inflammasome activation in response to adenoviruses. NALP3 and ASC are thought to function in the recruitment of caspase-1 to the inflammasome complex, which thereby leads to the maturation of pro-IL-1*β*. An established model of adenovirus infection in mice was used to demonstrate that NALP3, ASC and caspase-1 were all required for a maximal immune response to DNA viruses *in vivo* [95]. As evidence that the inflammasome response operates independently of other known DNA-sensing mechanisms, it was shown that, following exposure to adenovirus DNA, caspase-1 processing was intact in macrophages that lack both TLR9 and the TLR adaptor protein MyD88. Therefore, inflammasome activation is required *in vitro* and *in vivo* for mounting effective immune responses against DNA viruses, (e.g. Production of anti-DNA autoantibodies). It seems that the inflammasome response operates independently of other known DNA-sensing mechanisms.

Interestingly, the presence of nonviral DNA in the cytoplasm (of *Escherichia coli*, mammalian cells, or synthetically derived) also initiated inflammasome activation in THP-1 cells and mouse macrophages. However, such a response was not elicited by mammalian RNA, polyinosinic–polycytidylic acid or RNA viruses, demonstrating that inflammasome activation is specifically triggered by DNA [95]. It has been confirmed that, the inflammasome pathway is distinct and independent from the type I IFN response that is triggered following DNA recognition in the cytoplasm [95].Thus, in addition to identifying a new pathway for host and microbial DNA recognition, the studies described might provide clues to the pathology of autoimmune diseases such as systemic lupus erythematosus (SLE) and chronic arthritis, which are often associated with autoreactive antibodies specific for self DNA and increased levels of IL-1*β*. That such conditions might involve aberrant inflammasome function is a possibility that warrants further investigation [94]. Furthermore, clinical research studies have shown that anti-DNA autoantibodies isolated from patients with rheumatoid arthritis have significant pathogenic and clinical impact on the disease, while their only heavy and light chain variable single domains hydrolyze both double and single-stranded DNAs without sequence specificity [96, 97]. Given the fact that, very recently [72, 98] the free light and heavy chains with hydrolytic activity upon viral dsDNA have been discovered, as a part of so far nonidentified immune profile in sea turtle *Trachemyus scripta.* This discovery rises the question whether catalytic properties of anti-DNA antibodies have a broader, phylogenetic significance and the autoimmunity phenomenology is the creative, long-lasting product of the evolution across the phylogenetic tree [72, 98].

### 9.4. The Relevance of Pathogenic Anti-DNA Antibodie's Idiotypes

The most detailed experimental studies on this subject are coming from the group of Shoenfeld et al. It is impossible to analyze each step of this moiré then a decade-long research, but we shall mention the most illustrative examples that should fit into this context. Since the idiotypic network is an important mechanism for controlling the immune repertoire, [33, 36–38, 41, 47, 99] Shoenfeld et al. [100] have tested anti-idiotypic modulation employing concentrated specific natural polyclonal anti-double-stranded (ds) DNA anti-idiotypic antibodies obtained from a commercial IVIG in the treatment of experimental SLE. Specific natural polyclonal anti-dsDNA anti-idiotypic antibodies (IVIG-IDs) were affinity purified from IVIG on an anti-dsDNA±Sepharose column constructed from anti-dsDNA idiotypes (ID) affnity purified from 55 patients with active SLE [100]. NZB/W F1 mice were treated i.v. with 3 weekly injections of IVIG-ID (2 mg/kg/injection) or regular IVIG (400 mg/kg/injection) both before (age 8 weeks) and after developing anti-dsDNA antibodies at the age of 21 ± 22 weeks [100]. The IVIG-ID-treated mice showed a decline in the titer of anti-dsDNA antibodies during the treatment, reaching maximum suppression 1 week after the last injection. A significant difference in the proteinuria level in the IVIG-ID-treated group compared to the control group was observed. Immunohistology showed different patterns of IgG deposition, with mesangial and capillary wall deposits in controls and in the IVIG-treated group, but only mesangial deposits in the IVIG-ID-treated group. The survival time of the IVIG-ID-treated group was longer than the IVIG-treated group. Treatment with concentrated specific anti-dsDNA anti-ID prepared from commercial IVIG has been shown to be more effective in suppressing the humoral reaction and clinical signs of SLE than native IVIG. The authors concluded that those results point to the considerable regulatory role of anti-ID in the mechanism of action of IVIG in SLE [100].

Later on, in another approach of the same group, intravenous polyclonal immunoglobulins (IVIGs) were used as a therapy of autoimmune diseases and especially in conventional therapy resistance cases of SLE [40]. The main mechanism by which IVIG exerts its effect in autoimmunity entails the existence of specific anti-idiotypic antibodies in the compound against idiotypes of autoantibodies. Pathogenic idiotypes of autoantibodies were employed in the past to induce experimental autoimmune diseases via active immunization [40, 100]. In this study, a specific IVIG (sIVIG) was constructed from a commercial IVIGs absorption on an column of pathogenic idiotype and elution of the bound following immunoglobulins. It was found that the sIVIG was more effective than IVIG in suppressing murine SLE. Peptides obtained out from a library via the sIVIG were found to be effective in replacing the idiotypes of anti-DNA antibodies. sIVIG absorbed on a Id peptides column were found to be as effective in experimental murine SLE as sIVIG generated on an Id column. The “fished” Id peptides enable the commercial production of sIVIG [40]. In parallel, peptides derived from a pathogenic idiotype were also employed for therapeutical purposes in human phase I, II studies and suppressed SLE following active immunization. Thus, the idiotypic network proposed by Jerne in 1974 (as we already mentioned above) led to at least two novel therapeutic avenues in autoimmunity, by and large, and in SLE specifically [40]. Taken together, and in context of vaccine trials with specific idiotypes, these results indicate the necessity for deeper insight into possible mechanism of action of relevant idoitypic anti-DNA autoantibodies to enlighten their potential targeted therapeutic role in the spectrum of the entire mosaic of autoimmunity.

## 10. Take-Home Messages

- An essential criterion for the study of anti-DNA autoantibodies is their high purity.
- The novel magnetic bead-based method for isolation and purification of anti-ssDNA autoantibodies has been developed, thus enabling the study of this unique subset.
- Anti-DNA autoantibodies seem to have dual function: hydrolysis and cytotoxicity.
- Mouse monoclonal and human polyclonal antiss-DNA autoantibodies hydrolyze Gololobov's single stranded nucleotide sequence.
- The hydrolytic and cytotoxic activities of anti-DNA produce pathological symptoms during an attempt of the host to protect against the nucleic acids of microbes.
- Environmental factors such as some drugs, pristine, phthalates, infection, and some vaccines can alter the anti-DNA antibody response in mice and probably in humans.
- Some mechanisms of anti-DNA autoantibody secretion involve Toll-like receptor 9, inflammasomes, and DNA-sensing proteins.
- Idiotypic anti-DNA autoantibodies are pathogenic and can be used for vaccination.
- Drug-induced lupus does not induce production of anti-DNA autoantibodies.

## Figures and Tables

**Table 1:** Anti-DNA autoantibodies in SLE both belong to category of ANA 95%–98% Titer: 1 : 80 or lower is considered negative.

dsDNA	ssDNA
SPECIFICITY	SPECIFICITY
(i) Highly specific for SLE [18]	(i) NonSpecific [18]
(ii) Considered traditional markers of SLE	(ii) Rarely indicated
TEST SENSITIVITY	TEST-SENSITIVITY
(i) 60%	(i) 30%–70%
PRODUCERS (?)	PRODUCERS (?)
(i) 55%–75% of premature B-cells are prone to autoreactivity [18]	(i) CD5+/CD5*- *[39]
(ii) CD5+/CD5-[39]	(ii) RP105- [19]
(iii) RP105-[19]	(iii) RP105+ [19]
(iv) *X* = 19.5%	
(v) (Range: 8.8%–31%)	

**Table 2:** DNA recognition and binding by anti-DNA autoantibodies.

dsDNA	ssDNA
DNA-Recognition Mechanisms	DNA-Recognition Mechanisms
(i) Elusive	(i) Arginine involved [45]
DNA BINDING	DNA BINDING
(i) DNA phosphate backbone [61–63]	(i) Oligo-dT [17, 45]
(ii) Planted antigens [17]	(ii) Involvement of tryptophan and tyrosine in binding [17]
(iii) Cellular membrane proteins [65, 66]	DNA-binding MOTIFS
DNA-binding MOTIFS	(i) -cacc-caccc-accc-cccc blocks [60]
(i) 5′ gcg 3′/3′cgc5′motifs located in dsDNA [60].	

**Table 3:** Anti-DNA autoantibodies properties.

dsDNA antibodies	ssDNA antibodies
Ig class: IgG and IgM	Ig class: IgG and IgM
IgG subclass: IgG_3_	IgG subclass: IgG_1_ and IgG_3_
PATHOGENICITY: based upon binding criteria	PATHOGENICITY: based upon binding criteria
(i) Some forms of lupus nephritis	(i) Non-pathogenic?
(ii) CNS involvement	(ii) Pathogenic and nephrogenic in human and murine models
(iii) Correlates with disease activity	(iii) Predictors of lupus flares and anti-dsDNA increase in humans
Abzymes and DNA-hydrolytic activity	ABZYMES and DNA-HYDROLYTIC ACTIVITY
Human and mouse mono- and polyclonal	Mouse monoclonal. Human?
CYTOTOXIC ACTIVITY	CYTOTOXIC ACTIVITY
Human polyclonal	Undetermined⋯for now
